# Cholera in January!

**Published:** 1897-01-16

**Authors:** 


### CHOLERA IN JANUARY!

The arrival of cholera in England in the month of January shows how the course of epidemics and their modes of distribution are disturbed by modern developments of commerce. Ever since the great recrudescence of cholera which took place at the beginning of this century, the disease has tended again and again to escape from its endemic home in the Delta of the Ganges, and each time it has travelled along lines frequented by man and traversed by commerce. In 1826 it was carried by caravan routes through Central Asia to Russia, which country was also reached by another route by way of Persia. During 1831 it gradually traversed Europe, and in 1832, on reaching England, it quickly crossed the Atlantic and planted itself in America.

Again, both in 1848 and in 1854, it reached England travelling slowly by way of northern Europe.

In 1866, however, trade was drifting into new routes, and although the Suez Canal was not yet open, Egypt had become an important mercantile centre, and this time it was by Egypt that cholera was introduced into Europe.

Almost before this outbreak had died away another great wave of cholera had begun to flow over India, and spread by way of central Asia to Europe. As was its wont, it journeyed slowly, starting in 1867, and not reaching England till 1870.

In 1882 a new factor came into operation; the Suez Canal was in full working order, and Mecca, the centre of Mohammedan pilgrimages, became also the centre for the distribution of cholera.

In 1893 cholera again approached, and this time by the old northern route, by way of central Asia. But many things had happened in central Asia since the early days of cholera in 1830. A great railway system had gradually grown up, an iron band tying up the khanates of central Asia to their Russian master, and so it happened that although the cholera crept slowly, as it had done so many times before, through Afghanistan and north-eastern Persia, travelling slowly where man went slowly, it flew so soon as it touched the Trans-Caspian railway system. Baku was soon infected, the Trans-Caucasian railway carried it to the Black Sea ports, while the steamers on the Caspian took it up the Volga and placed it on the general railway system of European Russia. Within a month of the recognition of cholera at a town on the Trans-Caspian Railway it had penetrated to the heart of Russia, the transit having taken as many days as before the times of railways and steamboats it had taken months.

Whether the steamer "Nubia" has in the present instance brought the cholera with her all the way from Calcutta, or picked it up at Port Said or Colombo on the way, is perhaps a question. There is always plenty of cholera infection at Calcutta, and we hear of its recent presence in Colombo, and the existence of several cases of so-called "dysentery" among the Lascars, two of whom died while crossing the Indian Ocean, is suggestive of the possibility of the infection having been on board all the time. At the same time, it must be remembered that Egypt has for the last two years been under the same ban, and that cholera was certainly present there in great virulence last summer. It is reported from Cairo that there have been no cases in the country since September, but this must not be taken too seriously. It has been the complaint of the sanitary authorities all along that cases were concealed, and in view of the admittedly imperfect knowledge of the extent of the disease during last winter, it will not do to accept without reservation the denials of its existence now. Wherever it comes from, however, the lesson is plain that modern conditions of commerce leave us open to the importation of pestilential diseases from which we were protected in the past by the slowness of travel, and by the fact that no man could carry the infection far before being struck down by the disease. Everything that increases the speed of travel lengthens the striking distance of epidemics. Our great steamers, unless their sanitary arrangements are very perfect, are capable of carrying infection by long strides over great distances. They are like floating villages, or even towns, and infection may become endemic in them as it may among a community on land, so that while the first sufferers die and are cast overboard, the infection is not thus got rid of, but, by dint of passing through a series of victims, may be carried to the uttermost parts of the earth. Our old protection against such epidemics is broken down by modern commerce; let us hasten then to put on our new armour, which takes the form of modern sanitation. No longer can we hope to keep these diseases from our shores, but by good water, pure air, and clean living we may reasonably expect to render them harmless to our people.

